# Effects of liraglutide on ANP secretion and cardiac dynamics

**DOI:** 10.1530/EC-23-0176

**Published:** 2023-10-03

**Authors:** Shenghe Luo, Yunhui Zuo, Xiaotian Cui, Meiping Zhang, Honghua Jin, Lan Hong

**Affiliations:** 1 College of Pharmacy, Yanbian University, Yanji, China; 2 Department of Physiology and Pathophysiology, College of Medicine, Yanbian University, Yanji, China; 3 Department of Clinical Laboratory, Inner Mongolia Forestry General Hospital (The Second Clinical Medical School of Inner Mongolia University for Nationalities), Yakeshi, China; 4 Department of Cardiology, Yanbian University Hospital, Yanji, China; 5 Department of Pharmacy, Yanbian University Hospital, Yanji, China

**Keywords:** liraglutide, glucagon-like peptide 1 (GLP-1), atrial natriuretic peptide (ANP), PI3K/AKT/mTOR, piezo 1, cathepsin K, atrial dynamics

## Abstract

To observe the effects of liraglutide (analog of glucagon-like peptide 1 (GLP-1)) on atrial natriuretic peptide (ANP) secretion and atrial dynamics, an *ex vivo* isolated rat atrial perfusion model was used to determine atrial ANP secretion and pulse pressure. DPP-4^−/−^ mice were also established *in vivo*. ANP levels were determined by radioimmunoassay; GLP-1 content was determined by Elisa. The expression levels of GLP-1 receptor (GLP-1R), PI3K/AKT/mTOR, piezo 1, and cathepsin K were analyzed by Western blot. In the clinical study, patients with acute coronary syndrome (ACS) had low levels of plasma GLP-1 but relatively high levels of plasma ANP. In *ex vivo* (3.2 nmol/L) and* in vivo* (30 μg/kg) models, liraglutide significantly decreased ANP levels and atrial pulse pressure. Exendin9–39 alone (GLP-1R antagonist) reversibly significantly increased ANP secretion, and the reduction effect of liraglutide on the secretion of ANP was significantly alleviated by Exendin9–39. Exendin9–39 demonstrated slightly decreased atrial pulse pressure; however, combined liraglutide and Exendin9–39 significantly decreased atrial pulse pressure. Ly294002 (PI3K/AKT inhibitor) inhibited the increase of ANP secretion by liraglutide for a short time, while Ly294002 didn't counteract the decrease in pulse pressure by liraglutide in atrial dynamics studies. Liraglutide increased the expression of GLP-1R and PI3K/AKT/mTOR in isolated rat atria and the hearts of mice *in vivo*, whereas Exendin9–39 reversibly reduced the expression of GLP-1R and PI3K/AKT/mTOR. Piezo 1 was significantly decreased in wild type and DPP-4^−/−^ mouse heart or isolated rat atria after being treated with liraglutide. Cathepsin K expression was only decreased in *in vivo* model hearts. Liraglutide can inhibit ANP secretion while decreasing atrial pulse pressure mediated by GLP-1R. Liraglutide probably plays a role in the reduction of ANP secretion via the PI3K/AKT/mTOR signaling pathway. Piezo 1 and cathepsin K may be involved in the liraglutide mechanism of reduction.

## Introduction

Glucagon-like peptide 1 (GLP-1) is one of the components of incretin, which can regulate the metabolism of the body and has a wide range of pharmacological effects (1, 2). GLP-1R agonists like as liraglutide are currently widely used in the clinic as second-line therapy for type II diabetes, and GLP-1R agonists have been used in the clinical treatment of obesity in countries such as Korea and the United States (3, 4, 5). GLP-1R is expressed in many tissues, including the pancreas, liver, heart, gastrointestinal tract, and brain. GLP-1R agonists increase GLP-1 activity while protecting the heart from hypertension, myocardial hypertrophy, and myocardial fibrosis, suggesting that GLP-1R agonist therapy is not limited to diabetes and obesity (6, 7). GLP-1R is located in the atria of the heart and can regulate the secretion of various hormones.

Atrial natriuretic peptide (ANP) is a cardiac endocrine with a role in lowering blood pressure by regulating the water–salt balance in the body (8, 9). Also ANP is widely accepted as the diagnostic, prognostic, and cost-effective biomarkers for acute coronary syndrome (ACS) in the clinic (10). Stretch stimulation was known to be the strongest factor for ANP secretion (11). The proposal of the gut–heart GLP-1R–ANP axis links GLP-1R to ANP, and GLP-1 can play an important role in the secretion and expression of ANP (10). There was a positive correlation between GLP-1 and ANP secretion in non-diabetic studies with Angiotensinogen II-induced hypertension (11, 12, 13). This is in contrast to patient data from our previous clinical investigation, in which measurements of ANP and GLP-1 in patients with ACS showed a decrease in GLP-1 but an increase in ANP content. Therefore, we investigated the regulatory effect of GLP-1R agonist liraglutide on ANP secretion in the rodent heart and its mechanism.

## Materials and methods

### Patient samples

We collected 1409 patients aged between 25 and 80 years who attended the affiliated hospital of Yanbian University between 2021 and 2022. Among them, by the principle of patient voluntariness, 163 blood samples were collected for the determination of relevant indicators. The patients who do not have any cardiovascular disease are assigned to the control group. The rest patients were divided into the acute non-ST-elevation myocardial infarction (NSTEMI) group, acute ST-elevation myocardial infarction (STEMI) group, and unstable angina (UA) group according to myocardial injury index and ECG characteristics. Written informed consent for publication of their details was obtained from the patients involved in the study. The study protocol was approved by the Ethics Committee of Yanbian University Hospital, Yanji, Jilin Province, China, and informed consent was obtained from all the patients or the family members of the patients before the study.

### Animals

Sprague–Dawley (SD) rats (180 ± 40 g) and C57BL/6 wild-type (WT) mice (20 ± 5 g) were used, provided by the laboratory animal center of Yanbian University (License No. SCK (Ji)2003-0005) and Liaoning Changsheng Biotechnology Co., Ltd. (License No. SCXK (Liao)2020-0001). DPP-4 knockout mice (20 ± 5 g), purchased from Shanghai model organisms. DPP-4 inhibitors have been used in the treatment of type II diabetes mellitus (T2MD) to block the degradation and inactivation of GLP-1 (14). All animals, male and female, were kept in a clean, well-ventilated, and well-lighted environment with a temperature of 25–26℃ and a humidity of 70%. All animal experiments in this study were approved by the institutional animal protection and use committee of Yanbian University and followed the National Institutes of Health Guidelines for experimental animal protection.

### Preparation of a perfused beating rat atrium model *ex vivo*

SD rats were anesthetized i.p. with 0.4 mL/kg of 10% chloral hydrate. The left atrium of SD rats was fixed on a self-made device for atrial perfusion and placed in a glass culture dish containing 3 mL HEPES buffer. The left atrium of each rat was stimulated with electrical current (1.5 Hz, 0.3 ms, 30 V) and continuously supplied with oxygen at a temperature of 36℃. The HEPES buffer contained 118.0 NaCl, 4.7 KCl, 2.5 CaCl_2_, 1.2 MgCl_2_, 25.0 NaHCO_3_, 10.0 Glucose, 10.0 HEPES (pH 7.4 with NaOH), and 0.1% BSA (in units: mM).

### Experimental protocols and treatment reagents

Rats were randomized into five different groups: (i) control (HEPES buffer); (ii) liraglutide (3.2 nmol/L, Novo Nordisk); (iii) Exendin9-39 (0.3 nmol/L, Sangon Biotech, Shanghai, China); (iv) LY294002 (10 μmol/L, Sigma); and (v) Exendin9-39 + liraglutide (Exendin9-39 0.3 nmol/L, liraglutide 3.2 nmol/L).

### Experimental protocols

**Figure 1:**
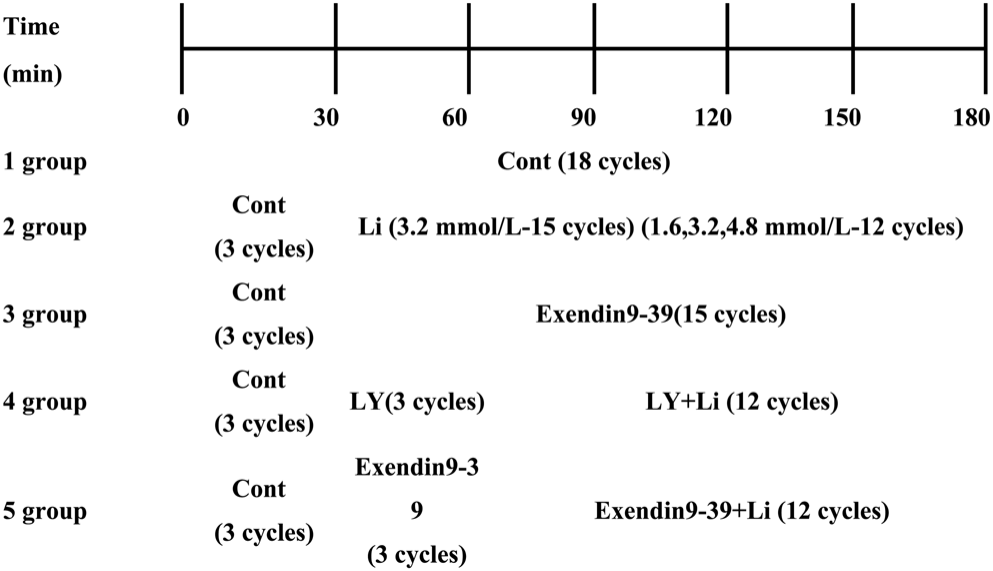
Experimental protocol for *in vivo* mouse experiments. 1 group: control group; 2 group: liraglutide group; 3 group: Exendin9-39 group; 4 group: LY294002 group. Cont (control, HEPES buffer); Li (liraglutide); LY (LY294002,PI3K inhibitor). Every 10 min is a cycle.

The left atrium was perfused according to five experimental groups (*n* = 6 for each group), as shown in Fig. 1. Every 10 min was an experimental cycle, and the peak value recorded in each cycle (10 min) was used as the atrial pulse pressure. Perfusion collected every three cycles (30 min) was stored at 4℃ for subsequent ANP measurement. At the end of the experiment, atrial tissue was removed and stored in liquid nitrogen for subsequent experiments.

### Preparation of mouse *in vivo* model

C57BL/6 wild-type and knockout mice (of both sexes) were used. Mice were divided into four groups (*n* = 6 for each group): (i) WT; (ii) WT + liraglutide; (iii) DPP-4^−/−^ (DPP-4 knockout mouse); and (iv) DPP-4^−/−^ + liraglutide. Normal saline (30 μg/kg) and liraglutide (30 μg/kg) were injected into WT mouse and DPP-4^−/−^ mouse by i.v., respectively, at same time every day for 14 days. After anesthesia with 0.2 L/min isoflurane, blood was inserted into the right ventricle and drawn into the ET tubes containing anticoagulants (10 μL EDTA and 20 μL aprotinin per ET anticoagulant tube) for subsequent ANP assays, the heart was immediately removed at −80°C for subsequent experiments.

### Determination of ANP content

ANP concentrations in plasma, perfusate, and atrial plasma were measured with iodine [125I] Atrial Natriuretic Factor Radioimmunoassay Kit (North Institute of Biological Technology, Beijing, China). ANP secretion is expressed in nanogram per minute per gram of atrial tissue wet weight (−80℃ was placed after anticoagulant treatment with aprotinin and EDTA in an ANP radioimmunoassay kit before collection of human, rat perfusate, and mouse plasma).

### ELISA

Plasma from 163 patients was subjected to ELISA experiments according to the instructions of the GLP-1 Elisa Kit (Shanghai Hengyuan Biotechnology Co., Ltd., China), and the absorbance values (OD values) were measured at 450 nm wavelength in combination with a microplate reader (Bio-Red). The concentration of GLP-1 was calculated according to the OD value of the sample.

### Western blot

At the end of the perfused experiment, the atrial muscle tissue or the apical tissue of the mouse model *in vivo* was treated with Ripa lysis, PMSF, and protein phosphatase inhibitor (Solarbio, China) according to the weight ratio. The tissue was homogenized and centrifuged by a tissue homogenizer, and then the BCA protein was quantified (Solarbio, China). After quantification, the samples were mixed with the appropriate amount of protein tracer loading buffer (Proteintech, China) and denatured. The proteins were separated by SDS-PAGE gel electrophoresis and then transferred to the PVDF membrane (Merck, USA). SDS-PAGE gel percentages were 8%, blotting membranes were blocked with 5% skim milk for 1 h and then incubated with the corresponding primary antibody overnight at 4℃, followed by incubation with the corresponding secondary antibody for 1 h at 25℃. After exposure with the enhanced chemiluminescence kit (CWBIO, China), the bands were processed with Image J software (National Institutes of Health, Bethesda, MD, USA). The following antibodies were used in this experiment: Anti-GLP1-1R Antibody (ab218532, Abcam), AKT (phospho-S473) polyclonal antibody(80455-1, Proteintech), Piezo 1-antibody(15939-1-AP, Proteintech), PI3 Kinase p85 (19H8) Rabbit mAb (4257S, CST, USA), T-AKT (60203-2, Affinity, China), Anti-β-Actin Antibody (BM3873, Boser, China), mTOR (7C10) Rabbit mAb(2983S, CST, USA), Phospho-mTOR (Ser2448) (D9C2) XP® Rabbit mAb (5536S, CST, USA), and anti-cathepsin K antibody (ab19027, Abcam).

### Statistical analysis

Experimental data were statistically processed with GraphPad Prism 9.0 (GraphPad Software; San Diego, USA), and all data are presented as mean ± s.e.m., and comparisons between two independent samples were performed using an unpaired *t*-test; comparisons between multiple groups were performed with one-way analysis of variance (ANOVA) and two-way ANOVA, and 0.05 was considered statistically significant.

## Results

### Changes of GLP-1 and ANP in the plasma of clinical patients

Plasma samples were collected from more than 160 clinical patients from 2021 to 2022 (provided by Affiliated Hospital of Yanbian University). Clinical studies have demonstrated that patients with ACS have high levels of ANP and BNP, and ACS is divided into STEMI, NSTEMI, and UA (15). There was no significant difference in the concentrations of ANP and GLP-1 among the three groups (STEMI, NSTEMI, and UA) (Table 1). The levels of GLP-1 in patients with ACS were significantly higher than those in controls (vs control, *P* = 0.0150; Table 1), but the levels of ANP and NT-proBNP in plasma were significantly lower than those in controls (vs control, *P* = 0.0002; Table 1), indicating the opposite effect of GLP-1 and ANP.

**Table 1:** Clinical patient’s plasma each index.

		Control (*n* = 16)	UA (*n* = 62)	STEMI (*n* = 64)	Non-STEMI (*n* = 21)	*P*
BMI (kg/m^2^)	BMI < 25	12 (75%)	31 (50%)	43 (67.2%)	13 (61.9%)	ns
	BMI ≥ 25	4 (25%)	31 (50%)	21 (32.8%)	8 (38.1%)	
Gender	Men	10 (62.5%)	29 (46.8%)	36 (56.3%)	13 (61.9%)	ns
	Women	6 (37.5%)	33 (53.2%)	28 (43.8%)	8 (38.1%)	
Age (years)	<65	14 (87.5%)	29 (46.8%)	28 (43.8%)	10 (47.6%)	0.0308
	≥65	2 (12.5%)	33 (53.2%)	36 (56.3%)	11 (52.4%)	
Diabetes mellitus (%)	N/A	4 (28.6%)	20 (32.3%)	22 (34.4%)	7 (33.3%)	ns
		12 (75%)	42 (67.7%)	42 (65.6%)	14 (66.7%)	
Hypertension (%)	N/A	3 (18.8%)	3 (35.5%)	32 (50%)	13 (61.9%)	ns
		13 (81.2%)	25 (64.5%)	32 (50%)	8 (38.1%)	
NT-proBNP (pg/mL)		68.45 ± 36.38	153.4 ± 367.4	4230 ± 5827^c,e^	2705 ± 5121^a,d^	<0.0001
Normalized ANP		26.12 ± 10.30	34.38 ± 18.81^a^	36.29 ± 15.83^b^	37.96 ± 26.52^a^	0.0150
Normalized GLP-1		293.6 ± 164.8	100.2 ± 108^b^	74.1 ± 65.96^b^	76.8 ± 40.69^b^	0.0002

Continuous data are presented as mean ± s.e. *P* < 0.05 is considered statistically significant. Values in bold:

^a^*P* < 0.05,^ b^*P* < 0.01, ^c^*P* < 0.001 vs control; ^d^*P* < 0.05, ^e^*P* < 0.001 vs UA.

NSTEMI, non-ST-segment elevation myocardial infarction; STEMI, ST-segment elevation myocardial infarction; UA, unstable angina.

### Effects of liraglutide on ANP levels *in vivo* and *ex vivo*

**Figure 2:**
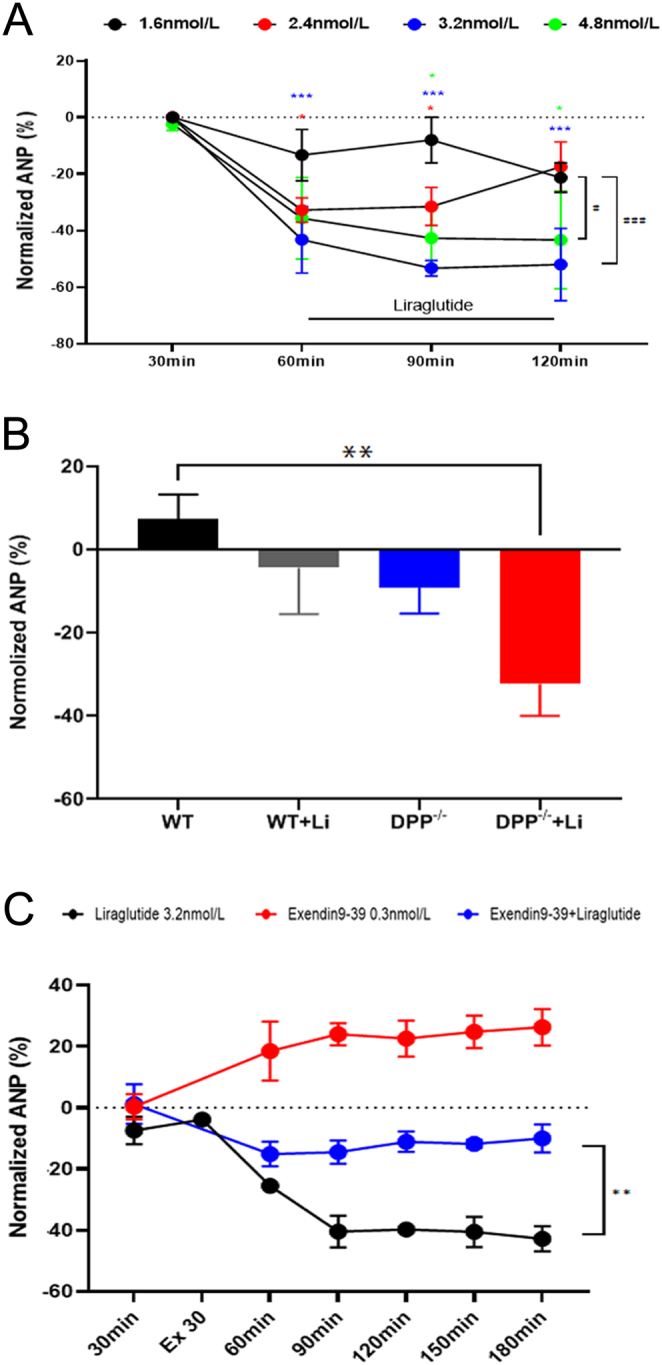
(A) Effects of different concentrations of liraglutide on the secretion of isolated rat atria. (B) Effect of liraglutide on ANP concentration* in vivo.* (C) Effect of liraglutide, Exendin9-39, and Exendin9-39 + liraglutide on ANP secretion in isolated rat atria. Exendin9-39 (GLP-1 antagonist, 0.3 nmol/L); WT: WT (wild- type) mouse + saline (30 μg/kg); WT + Li (liraglutide) (30 μg/kg); DPP-4^−/−^ : DPP-4^−/−^ mouse (DPP-4 knockout mouse) + saline (30 μg/kg); DPP-4^−/−^ + Li: DPP4^−/−^ mouse + liraglutide (30 μg/kg). (A) ^*^P < 0.05, ^***^P < 0.001 vs control *ex vivo*; (B) ^**^P < 0.01 vs WT *in vivo*; (C) ^**^P < 0.01 vs liraglutide *ex vivo* (mean ± s.e., *n* = 6).

In the atria of isolated rats, liraglutide can reduce the secretion of ANP at the concentration of 1.6, 2.4, 3.2, and 4.8 nmol/L, and the average ANP concentration is the lowest at the concentration of 3.2 nmol/L (vs control, ^***^P < 0.001; Fig. 2A and Supplementary Fig. 1, see section on supplementary materials given at the end of this article). *In vivo*, however, ANP concentrations were reduced in DPP-4^−/−^ mice compared with WT, while liraglutide decreased ANP concentrations in WT and DPP-4^−/−^ mice, only significantly in DPP-4^−/−^ mice (vs WT, ^*^P < 0.05; Fig. 2B). To further verify that liraglutide can reduce the concentration of atrial ANP secretion, Exendin9-39 (GLP-1R antagonist) was used. Exendin9-39 significantly increased atrial ANP secretion in isolated rats. In addition, the reduction effect of liraglutide on the secretion of ANP was significantly alleviated when treated with Exendin9-39 (vs liraglutide,^ **^P < 0.01; Fig. 2C).

### Effect of liraglutide on GLP-1R expression

**Figure 3:**
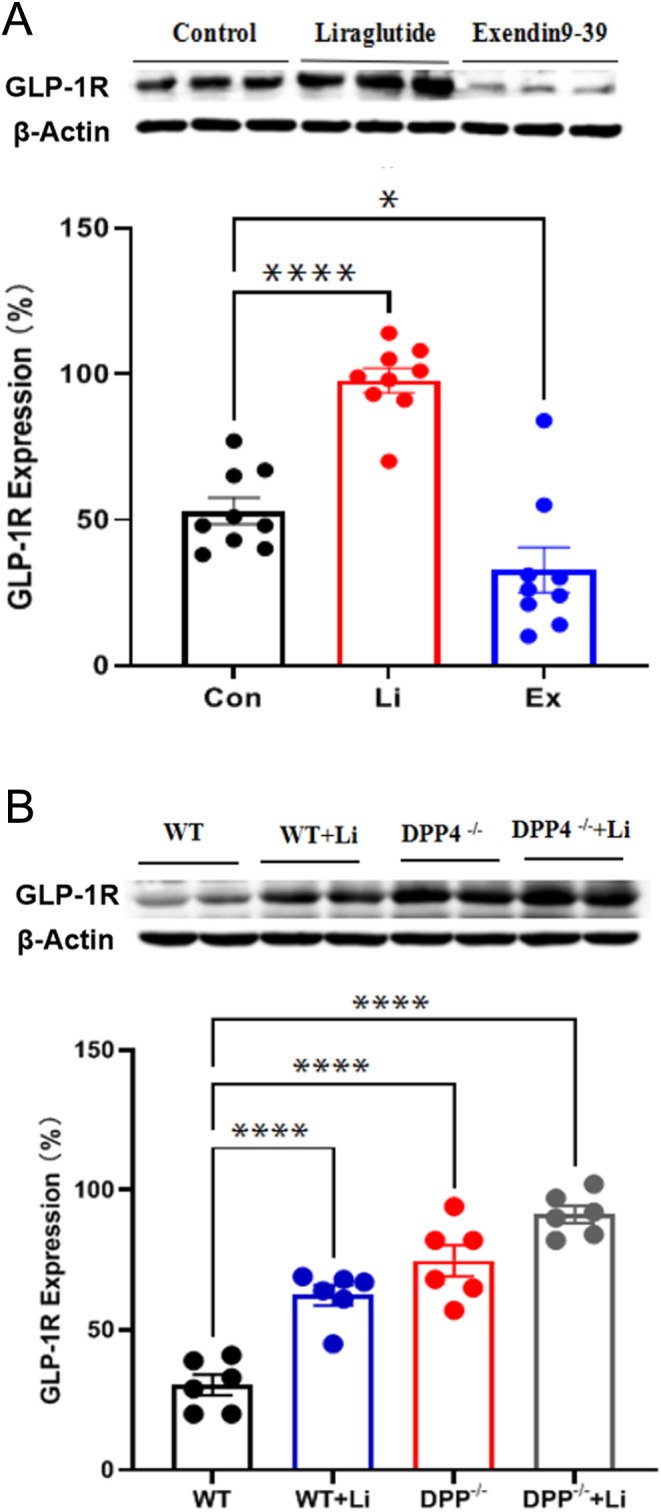
Expression of GLP-1R in atrial tissues by liraglutide *ex vivo* (A) and *in vivo* (B). Control, control group; Li, liraglutide: GLP-1R agonists, 3.2 nmol/L; Exendin9-39: GLP-1R inhibitors, 0.3 nmol/L. WT, WT (wild-type) mouse + saline (30 μg/kg); WT + Li, WT mouse + liraglutide (30 μg/kg); DPP-4^−/−^ , DPP-4^−/−^ mouse + saline (30 μg/kg); DPP-4^−/−^ + Li, DPP-4^−/−^ mouse + liraglutide (30 μg/kg). ^*^P < 0.05, ^****^P < 0.0001 vs control *ex vivo*; ^****^P < 0.0001 vs WT *in vivo*. (mean ± s.e., *n* = 6).

Western blot showed that liraglutide significantly increased GLP-1R expression in isolated rat atria (vs control, ^****^P < 0.0001; Fig. 3A), and increased the expression of GLP-1R in both WT and DPP-4^−/−^ mouse hearts (vs WT, ^****^P < 0.0001; Fig. 3B). Inversely, Exendin9-39 significantly reduced GLP-1R expression in rat atria (vs control, ^*^P < 0.05; Fig. 3A). These results illustrated GLP-1R was present in rat and mice hearts, also liraglutide plays roles through GLP-1R.

### Effects of PI3K/Akt/mTOR signaling pathways on liraglutide-inhibiting ANP secretion

**Figure 4:**
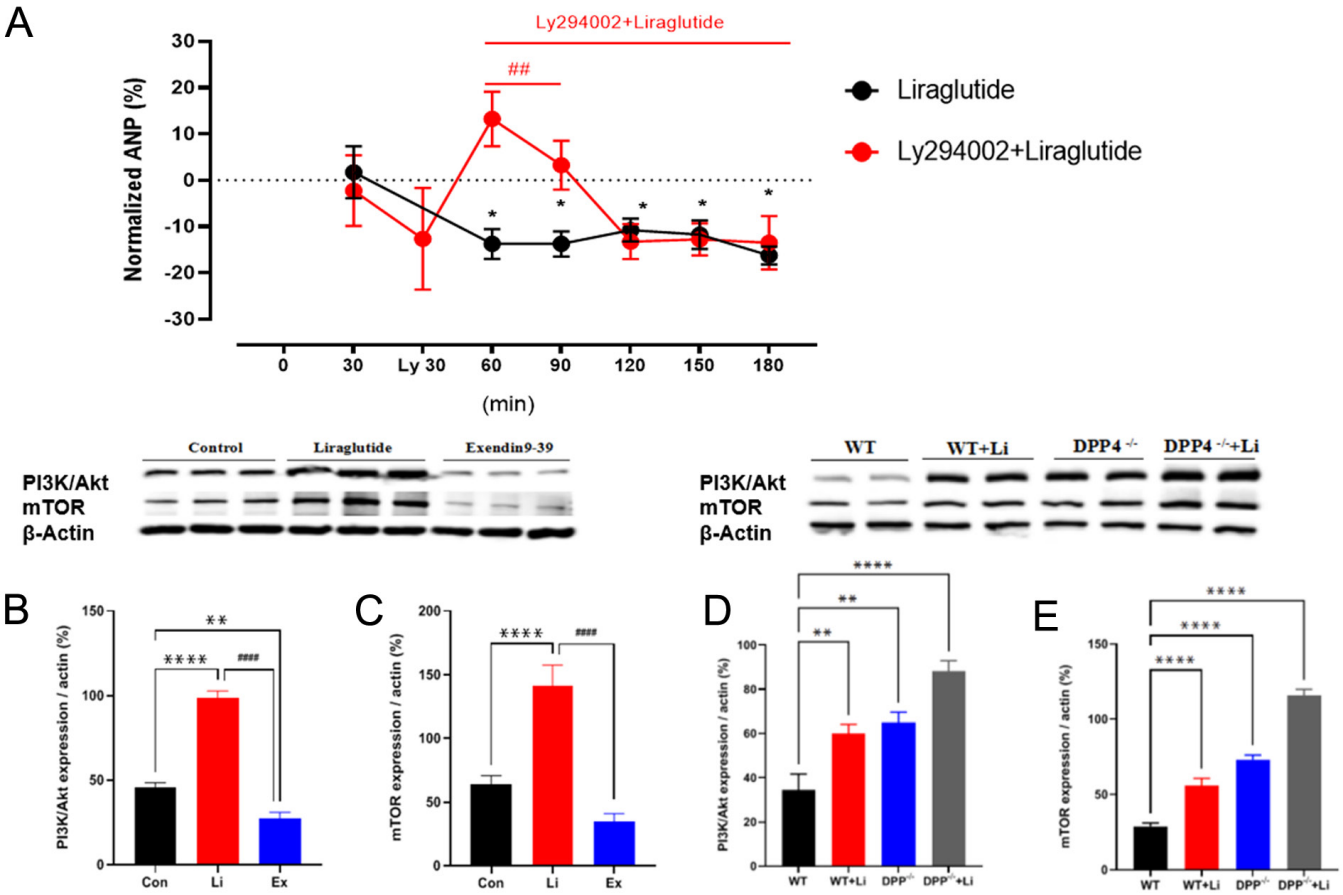
(A) Effect of LY294002 on atrial ANP secretion.Effects of liraglutide on the expression of PI3K/AKT/mTOR in isolated rat atria (B, C) and *in vivo* mouse hearts (D, E). Con, control group; Li, liraglutide: GLP-1R agonist, 3.2 nmol/L; Exendin9-39: GLP-1R antagonist, 0.3 nmol/L. WT, WT (wild-type) mouse + saline (30 μg/kg); WT + Li, WT mouse + liraglutide (30 μg/kg); DPP-4^−/−^, DPP-4^−/−^ mouse (inhibiting consumption of endogenous GLP-1) + saline (30 μg/kg); DPP-4^−/−^ + Li, DPP-4^−/−^ mouse + liraglutide (30 μg/kg). ^**^P < 0.01, ^****^P < 0.0001 vs control *ex vivo*; ^**^P < 0.01, ^****^P < 0.0001 vs WT* in vivo*;^ ##^*P* < 0.01, ^####^*P* < 0.0001 vs liraglutide *ex vivo* (mean ± s.e., *n* = 6).

The GLP-1R couples to multiple G proteins, leading to the activation of several intracellular signaling pathways (144), including the activation of PI3K/Akt/mTOR signaling pathways (16, 17). Therefore, We first investigated whether PI3K/AKT/mTOR signaling pathway was involved in liraglutide inhibiting ANP secretion. The results showed that LY294002 (PI3K inhibitors) could resist the inhibition of liraglutide on the secretion of ANP at the first three cycles (vs liraglutide 30–60 min, ^##^*P* < 0.01; Fig. 4A); however, after six cycles, LY294002 gradually reduced the inhibitory effect of liraglutide on ANP secretion. Western blot results showed that liraglutide significantly upregulated the expression of PI3K/AKT and mTOR in both *in vivo* heart and isolated rat atria (vs control, ^****^P < 0.0001; vs WT mouse, ^**^P <0.01, ^****^P < 0.0001; Fig. 4B, C, D, andE). In contrast, Exendin9-39 downregulated the expression of PI3K/Akt and mTOR in isolated rat atria (vs liraglutide, ^####^*P* < 0.0001; Fig. 4B and C). The results initially verified that liraglutide inhibited ANP secretion via PI3K/AKT/mTOR signaling pathway.

### Effects of liraglutide on atrial dynamics in isolated rat atria

**Figure 5:**
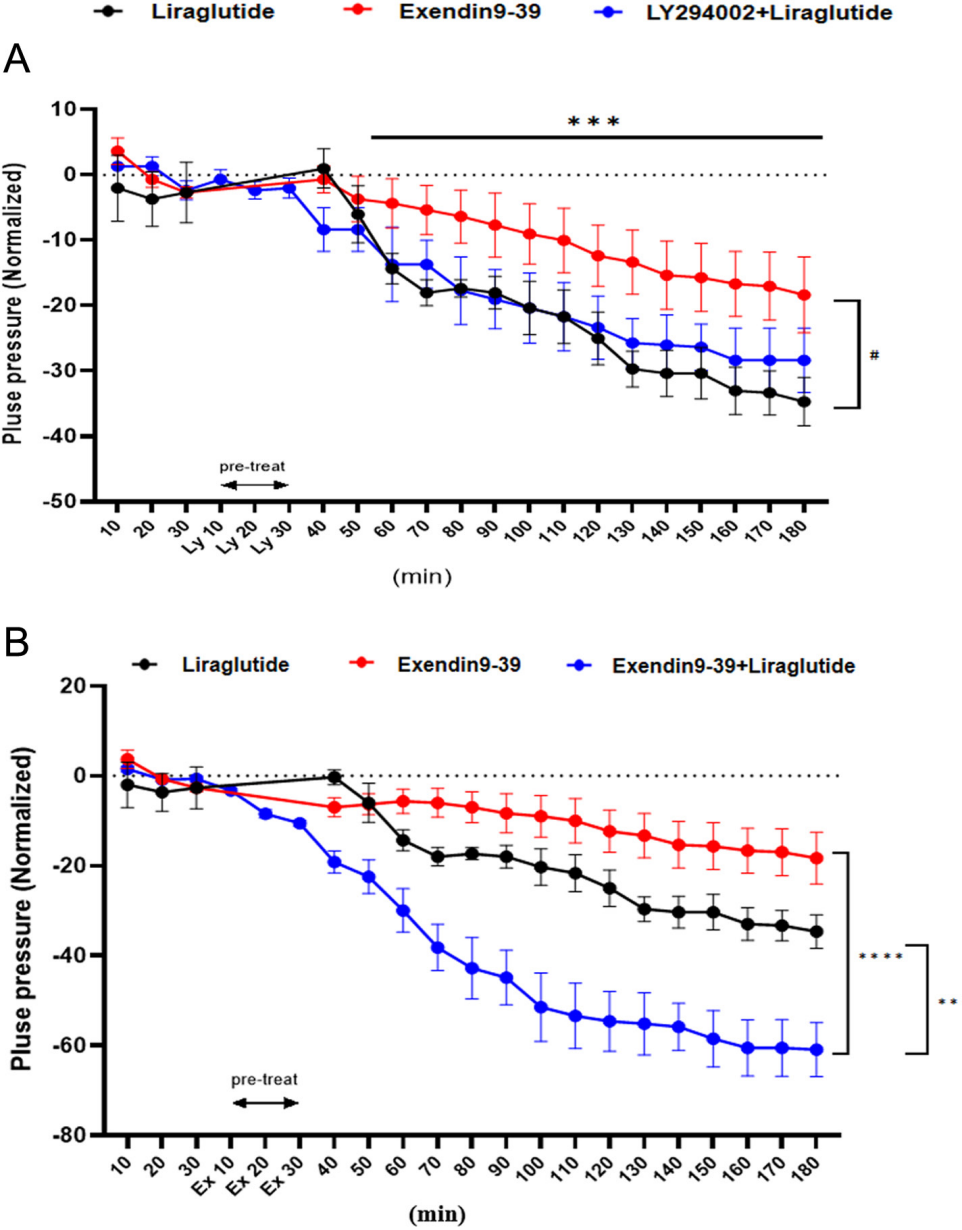
Effect of liraglutide, Exendin9-39, LY294002 (A), and Exendin9-39 + liraglutide (B) on mechanical activity of isolated rat atria. Liraglutide: GLP-1R agonist, 3.2 nmol/L; Exendin9-39: GLP-1R antagonist, 0.3 nmol/L; LY294002: PI3K inhibitor, 10 μmol/L; Exendin9-39 + liraglutide: Exendin9-39 0.3 nmol/L, Liraglutide 3.2 nmol/L. (A) ^#^*P* < 0.05 vs liraglutide *ex vivo*; ^***^P < 0.001, vs control in 60–180 min *ex vivo*. (B) ^**^P <0.01, ^****^P < 0.0001 vs Exendin9-39 + liraglutide *ex vivo* (mean ± s.e., *n* = 6).

Liraglutide could reduce atrial pulse pressure (vs control in 60–180 min *ex vivo*, ^***^P < 0.001; Fig. 5A) as well as Exendin9-39 also decreased atrial pulse pressure (vs liraglutide, ^#^*P* < 0.05; Fig. 5A). However, atrial pulse pressure was more significantly reduced when treated with Exendin9-39 and liraglutide together, compared with Exendin9-39 or liraglutide alone (vs Exendin9-39 + liraglutide, ^**^P <0.01, ^****^P < 0.0001; Fig. 5B). This results showed that the lowering effect of Exendin9-39 on atrial pulse pressure is independent of liraglutide probably, Exendin9-39 itself may affect atrial pulse pressure through other ways that we have not yet discovered. In addition, LY294002 did not reverse the effect of liraglutide on reducing atrial pulse pressure. The results preliminarily confirmed that the regulation of atrial pulse pressure by liraglutide is related to GLP-1R but may not be related to PI3K.

### Effects of liraglutide on expression of piezo 1 and cathepsin K

**Figure 6:**
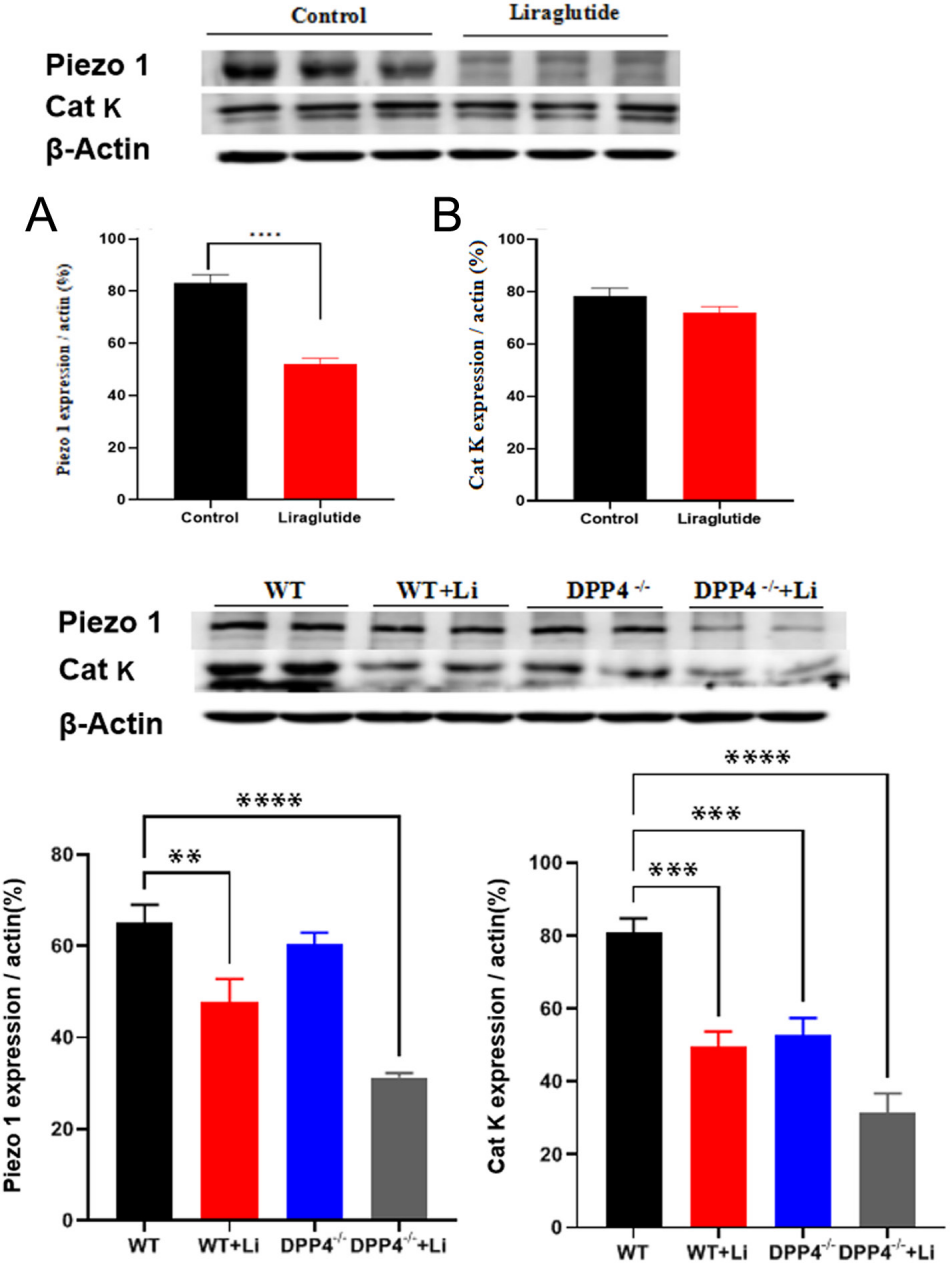
Effects of liraglutide on the expression of calcium regulatory proteins piezo 1 and cathepsin K *in vivo* (A, B) and *ex vivo* (C, D). Con, control group; Li, liraglutide: GLP-1R agonist, 3.2 nmol/L. WT, WT (wild-type) mouse + saline (30 μg/kg); WT + Li, WT mouse + liraglutide (30 μg/kg); DPP-4^−/−^, DPP-4^−/−^ mouse (inhibiting consumption of endogenous GLP-1) + saline (30 μg/kg); DPP-4^−/−^ + Li, DPP-4^−/−^ mouse + liraglutide (30 μg/kg). ^****^P < 0.0001 vs control *ex vivo*; ^**^P < 0.01, ^***^P < 0.001, ^****^P < 0.0001 vs WT* in vivo* (mean ± s.e.,* n* = 6).

Piezo1 is a mechanosensitive ion channel protein in humans (18). Calcium regulation of atrial myocytes plays an important role (19). Cathepsin K is known to be involved in calcium homeostasis (20). Results showed that piezo 1 and cathepsin K were significantly decreased *in vivo* in mice (WT mouse and DPP-4^−/−^ mouse), and liraglutide can downregulate the expression of piezo 1 and cathepsin K in rat atria and mouse hearts *in vivo* but not in liraglutide rat atria *ex vivo* (vs control, ^**^P < 0.01; vs WT, ^**^P < 0.01, ^***^P < 0.001, ^****^P < 0.0001; Fig. 6A, B, C, andD). Cathepsin K expression was significantly reduced in DPP-4^−/−^ mice hearts *in vivo* but not in piezo 1 (vs WT, ^***^P < 0.001; Fig. 6C and D). These results suggest that cathepsin K and piezo 1 may be involved in the regulation of liraglutide mechanical activity.

## Discussion

GLP-1, an incretin-like peptide that regulates blood glucose by stimulating insulin secretion and inhibiting glucagon, is currently widely used in the treatment of T2MD (1, 21). In some countries, GLP-1 has been applied to the long-term treatment of clinical obesity, and GLP-1 can promote nerve growth and reduce neuroinflammation, which has become a hotspot in the research of neurodegenerative therapy (21). In recent years, an increasing number of studies have found that GLP-1 also plays an important role in the cardiovascular system and can prevent the occurrence of diseases, with potential cardioprotective effects (21, 22). There have been studies showing lower levels of GLP-1 in patients with ACS, which is consistent with our pre-experimental findings (23). ANP is a hormone secreted by the heart and is involved in the treatment of many cardiovascular diseases and the maintenance of cardiovascular homeostasis (24). In our study, increased ANP secretion was shown in patients with ACS, which seems to indicate an antagonistic effect between GLP-1 and ANP. In our animal experiments, it has also been confirmed that the secretion of ANP has traditionally been inhibited. However, some studies have shown that GLP-1 can promote the secretion of ANP, and the reason for the different results may be due to the different experimental methods and doses (12).

**Figure 7:**
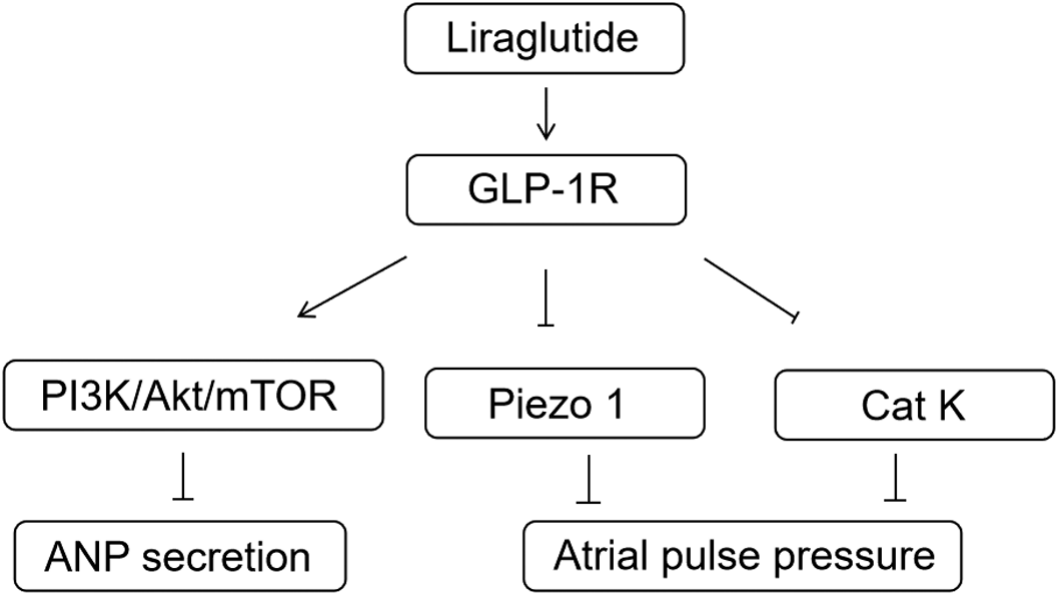
Possible mechanisms by which liraglutide inhibits atrial natriuretic peptide secretion and atrial dynamics.

GLP-1 receptor (GLP-1R) can be activated by GLP-1 agonists to regulate blood glucose, reduce body weight, and lower blood pressure (17, 25, 26, 27). GLP-1R is a class of G protein-coupled receptors that can be activated by polypeptide agonists and is an effective therapeutic target for a variety of diseases (28, 29, 30, 31). Ligands binding liraglutide to GLP-1R involve a variety of signal cascades, including PI3K, AKT, mTOR, and PKA (32, 33, 34). Studies have shown that GLP-1 reduces myocardial infarction size and heart failure through PI3K/Akt (35, 36). We investigated the effects of liraglutide on ANP secretion, which decreased ANP secretion in isolated rat atria and *in vivo* mouse hearts and was inhibited by LY294002 (PI3K inhibitor) and Exendin9-39 (GLP-1R antagonist). Moreover, DPP-4 knockdown can increase the decrease of liraglutide on ANP secretion. At the same time, liraglutide increased the expression of PI3K/Akt and mTOR in isolated rat atrium and mouse hearts. This suggests that liraglutide activates GLP-1R to reduce ANP secretion possibly through PI3K/Akt/mTOR (Fig. 7).

Liraglutide has been reported to regulate heart rate (37) and to lower blood pressure (38). The decrease in heart rate and blood pressure is the result of the negative inotropic of the heart, and the contraction of the heart muscle is also the result of the negative inotropic, so there may be a link between liraglutide and myocardial contraction (39, 40, 41). We found that liraglutide reduced atrial pulse pressure, but Exendin9-39 still reduced atrial pulse pressure. However, there was a more significant reduction in atrial pulse pressure when Exendin9-39 was treated with liraglutide. Therefore, we speculate that Exendin9-39 itself has a decreasing effect on atrial pulse pressure in this experimental model, and the effect of Exendin9-39 on atrial pulse pressure may be independent of the effect of liraglutide on atrial pulse pressure. Exendin9-39 itself may affect atrial pulse pressure in other way that we have not yet discovered. The effect of Exendin9-39 itself on the mechanical activity of the heart needs to be further studied. Cathepsin K and piezo 1 play an important role in regulating Ca^2+^ balance (18, 19, 20, 42). Cathepsin K can regulate calcium homeostasis *in vivo* by regulating calcineurin, in which Ca^2+^ is involved in cardiac excitation–contraction coupling and affects cardiac mechanical activity (18, 19, 20, 42). Our results showed that liraglutide reduces atrial pulse pressure, suggesting that liraglutide may affect atrial mechanical activity by regulating calcium homeostasis through cathepsin K (40, 41). Piezo 1 is a typical mechanosensitive ion channel protein that links mechanical forces to biological signals (42). Mechanical stimulation of bone and nerve cells increases the expression of piezo 1, which promotes bone and nerve growth through biological signals (43, 44, 45). However, other studies have shown that piezo 1 regulates cell response to mechanical stress by controlling Ca^2+^ influx, and that piezo 1 expression increases in red blood cells and hepatic portal endothelial cells when subjected to mechanical forces. Calcium ion inflow increases through calcium osmotic channels (42, 46, 47, 48). Our results showed that liraglutide decreased atrial pulse pressure and piezo 1 expression. It is not clear whether liraglutide regulates the Ca^2+^ influx through the decreased expression of piezo 1, which reduces atrial pulse pressure, or whether liraglutide first causes changes in the atrial pulse pressure, which decreases the expression of piezo 1. Whether liraglutide reduces the atrial pulse pressure or the expression of piezo 1 first remains to be studied.

At present, the incidence of myocardial diseases in diabetic patients is increasing, and diabetic cardiomyopathy has been paid more and more attention (49). Studies have shown that liraglutide and ANP have protective effects on diabetic cardiomyopathy, liraglutide has protective effects on myocardial infarction and hypertrophy in diabetic mice, and exogenous ANP can improve myocardial injury caused by ischemia reperfusion in diabetic mice (50, 51, 52). However, the effect of liraglutide on cardiac endocrine and its mechanism are still unclear. Therefore, we studied the effects of liraglutide on cardiac endocrinology through *in vivo* and *ex vivo* experiments, which will play a guiding role in the clinical treatment of diabetes and diabetic cardiomyopathy.

## Supplementary Materials

Supplementary Figure 1

Supplementary Figure 2

Supplementary Figure 3

## Declaration of interest

The authors declare that there is no conflict of interest that could be perceived as prejudicing the impartiality of the research reported.

## Funding

This work did not receive any specific grant from any funding agency in the public, commercial, or not-for-profit sector.
