# Supplementary figures and images for: Effects of liraglutide on ANP secretion and cardiac dynamics
Source: Endocr Connect. 2023 Oct 3;12(11):e230176. doi: 10.1530/EC-23-0176 (PMC10563649; doi:10.1530/EC-23-0176)

Normalized ANP (%)

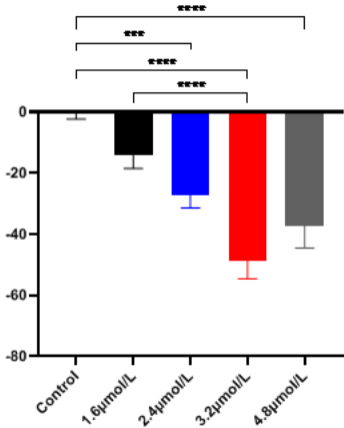

Supplement: Supplementary Figure 1 [file supplementary_figure_1.pdf]

● Liraglutide 3.2nmol/L

● Exendin9-39 0.3nmol/L

● Exendin9-39+Liraglutide

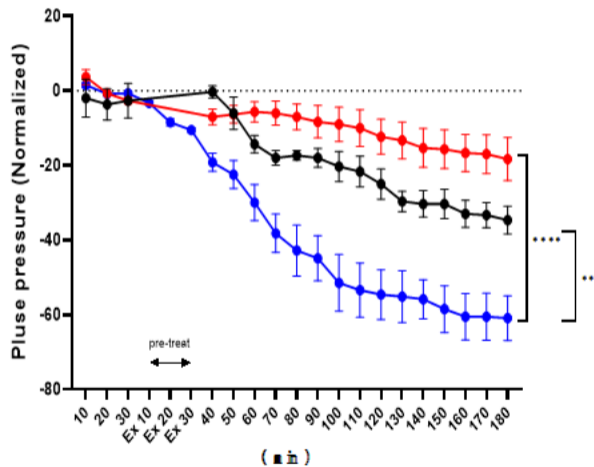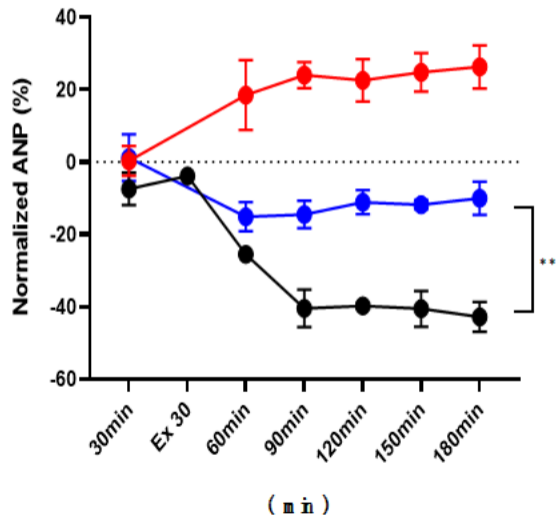

Supplement: Supplementary Figure 2 [file supplementary_figure_2.pdf]

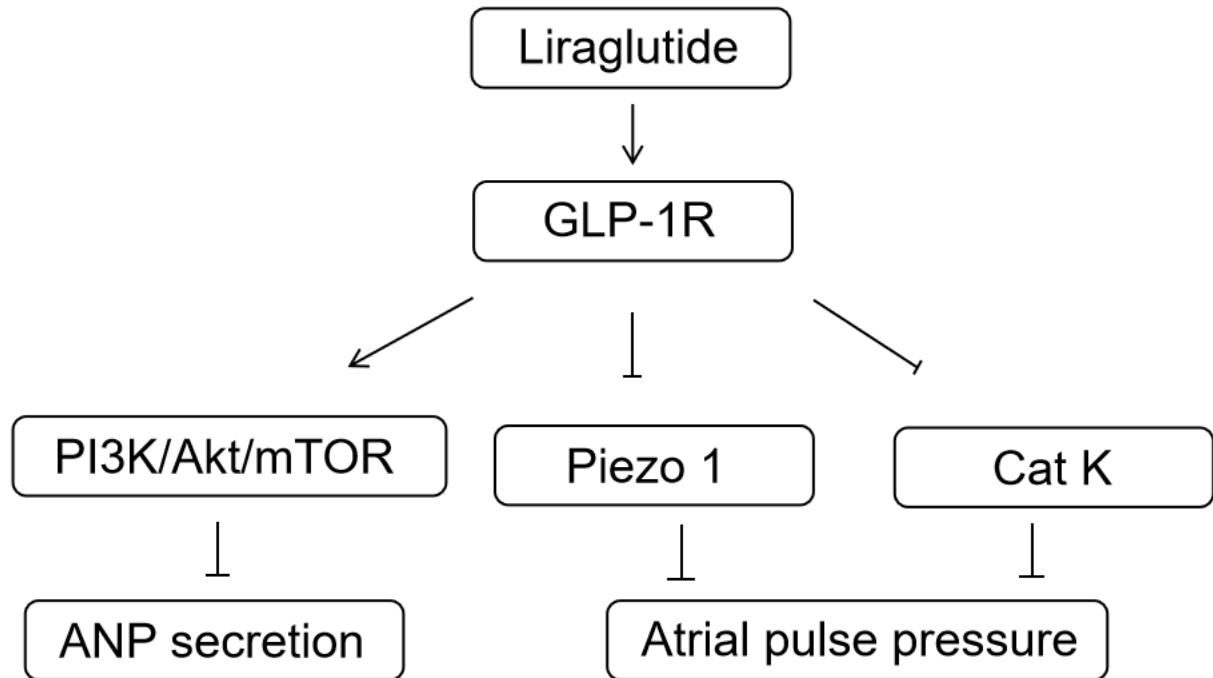

Supplement: Supplementary Figure 3 [file supplementary_figure_3.pdf]
